# The Prevalence and Severity of Tooth Wear in Type 2 Diabetic Patients

**DOI:** 10.1155/2018/3608158

**Published:** 2018-12-11

**Authors:** Patcharawan Srisilapanan, Matee Jindarat, Jeffrey Roseman

**Affiliations:** ^1^Center of Excellence in Dental Public Health, Faculty of Dentistry, Chiang Mai University, Chiang Mai, Thailand; ^2^Wiang Pa Pao Community Hospital, Wiang Pa Pao, Chiang Rai, Thailand; ^3^Professor Emeritus, Department of Epidemiology, UAB School of Public Health, University of Alabama, Tuscaloosa, Alabama, USA

## Abstract

**Objective:**

To assess the prevalence and severity of tooth wear in type 2 diabetic patients.

**Methods:**

Attendees at a diabetic clinic at Wiang Pa Pao Hospital in Chiang Rai province, Thailand, were invited to take part in this cross-sectional study. All participants were aged 35–74 and had type 2 diabetes. Participants were required to have been diagnosed with diabetes for at least three months. 179 subjects accepted a clinical oral examination and completed the questionnaire. Tooth wear was assessed clinically using the Smith and Knight Tooth Wear Index.

**Results:**

The mean age of diabetic patients was 56.5 ± 7.8 years. The majority (44.1%) had diabetes more than 5 years. The average years of having had diabetes was 6.5 ± 6.3 years. The most prevalent type of tooth wear was attrition (99.4%). The prevalence of erosion, abrasion, and abfraction were 64.8%, 31.3%, and 7.3%, respectively. The majority of the tooth wear was moderate to high severity (62.1%). Erosion and abfraction showed significant association with age group (*p* < 0.05). Age group was significantly associated with the severity level (*p*=0.017). Mild tooth wear severity was the highest in age groups 35–44 and 45–54 (53.8% and 41.2%, respectively). Moderate tooth wear was the highest proportion in age groups 55–65 and 65–74 (52.2% and 44.0%, respectively). There were no significant differences between specific diabetic symptoms and types of tooth wear.

**Conclusion:**

There was a high prevalence of tooth wear among diabetic patients. The role of prevention is vital in maintaining the integrity of the teeth and to avoid treating these worn teeth in diabetic patients.

## 1. Background

Older people are living longer and maintaining more natural teeth. Functional teeth are important for eating and enable older people to consume normal and healthy food. However, natural tooth retention is negatively affected by tooth wear. Tooth wear has recently been accepted as a major oral health problem [1–3]. Tooth wear is a progressive condition that affects dentition throughout life. Tooth wear affects both function and esthetics. The most important function affected is eating as worn teeth cannot be effectively used to bite and chew food [4]. Therefore, older patients suffer and seek dental treatment related to tooth wear.

Tooth wear includes attrition, abrasion, erosion, and abfraction. Attrition is the loss of tooth occlusal surfaces due to the force of tooth against tooth. Abrasion is the wear on buccal surfaces related to forces mainly caused from tooth brushing. Erosion is the loss of dental hard tissue from combined chemical-mechanical forces related to acids from diet [5]. Treatment of excessive tooth wear is complicated, expensive, and time-consuming. A study on the cost of prosthodontic rehabilitation of erosive tooth wear for National Health Service hospitals in the United Kingdom confirmed that rehabilitation is complex, interdisciplinary, and costly [3]. People in rural areas who are poor may not be able to afford sophisticated treatments such as crowns and bridges.

There are several extrinsic factors related to different types of tooth wear [5–9]. Extrinsic factors for dental erosion are commonly found to be related to diet and dietary behaviors [5]. Acid in one's diet is the main cause of erosion. Common dietary acids associated with acid erosion are found in citrus fruits or fruit juices, carbonated drinks, wine, and vinegar [5–7]. Factors related to tooth attrition included bruxism, chewing patterns, etc [8]. Abrasion is usually associated with tooth brushing forces and pattern and abrasive particles in toothpastes [9].

Tooth wear can be found in all age groups. However, it is more prevalent and more severe in adults and older people [10–13]. Yadav [11] studied 500 subjects aged 18 to 55 in India. He found a high prevalence of tooth wear (88%) in older people. A study of 704 adults in China found more than 80% of tooth wear in premolar and molar teeth [12]. In Dutch adult population, the prevalence of moderate tooth wear was 80% [13]. In Thailand, there are very few studies on tooth wear. There have been studies on tooth wear in teenagers [14] but no studies on tooth wear in older adult groups.

Diabetes is one of the most common noncommunicable diseases found in older people [15]. Over 60% of people with diabetes worldwide live in Asia, with prevalence across countries ranging from 3% to 47.3%. There is a high prevalence of diabetes in Thailand [15]. Data from the National Health Examination Survey (NHES) of Thailand in 2004, 2009, and 2014 reported an increase over time in the age-adjusted prevalence of diabetes in individuals aged 20 and above. The increase ranged from 7.7% in 2004 to 7.8% in 2009 and 9.9% in 2014. The prevalence of diabetes in those who were 60 years old and above in 2014 was 15.3% in males and 20.3% in females [16]. It is therefore considered as a major public health problem. Diabetic patients are one important target groups for oral health care under the policy of the Thailand Ministry of Public Health. Diabetic patients are among the largest group for dentists to apply oral health prevention and promotion to achieve good oral and overall health. The primary care services at all community hospitals in Thailand give high emphasis on patients with diabetes. Dental practitioners should be aware of the prevalence and severity as well as the underlying factors related to tooth wear. It is therefore worthwhile to investigate the prevalence and severity of tooth wear among diabetic patients in northern Thailand.

## 2. Materials and Methods

This cross-sectional, descriptive, and analytic survey was conducted in Wiang Pa Pao District, Chiang Rai province, Northern Thailand. The total number of diabetic patients at Wiang Pa Pao hospital in 2012 was 1,911. Using quota sampling according to age groups, the calculation of the sample size was 150. 179 subjects participated in this study. Diabetic patients were identified by the medical doctor as those who had fasting blood sugar (FBS) ≥ 140 at least once among three consecutive blood sample screenings during their three-month period of hospital visits. These patients attended the diabetic clinic at the Wiang Pa Pao Community Hospital and Health Promoting Hospitals in Wiang Pa Pao District.

## 3. Data Collection

In this study, the term tooth wear includes attrition, erosion, abrasion, and abfraction. This study used the classification of tooth wear and the severity score according to Smith and Knight Tooth Wear index [17]. Smith and Knight Tooth Wear Index (TWI) is a worldwide acceptable and widely used index to assess tooth wear [13, 18, 19]. TWI is good for diagnostic purposes. It could assess the amount of the surface area involved and the depth of the surface loss [19]. One other advantage of this index is that it could determine the severity of wear on each surface. Each tooth was recorded for wear on all four surfaces (buccal, cervical, lingual, and incisal-occlusal), irrespective of the etiology of tooth wear. Only one diagnostic was given for each tooth.

One dentist (MJ) examined all subjects. The examiner was trained the use of Smith and Knight Index with the expert who was a professor in a dental school prior to the examination. Intraexaminer reliability was performed during the examination. Every 10 subjects examined, one subject would be re-examined which covered 10 percent of the total subjects. 18 subjects were included in the intraexaminer reliability from the total 179 subjects. Cohen's kappa coefficient was 0.88 which represented high reliability. Patients were examined in the dental clinic at Wiang Pa Pao Community Hospital. Data were collected during a three-month period from June to August 2013. Descriptive statistics, a chi-squared test, and a Fisher Exact test were used for data analysis.

## 4. Questionnaire

A face to face interview was performed by two dental nurses. The nurses were trained by a qualified dentist. The questionnaire was developed by the authors (PS and MJ) to include demographic information and related health factors. Content validity of the questionnaire was performed by three dental public health experts. The questionnaire was pretested in 30 subjects who had similar characteristics with the study subjects.

## 5. Results

The majority (69.9%) of the subjects were female. The mean age was 56.5 (±7.8). Almost half (44.1%) of diabetic cases had diabetes more than five years. The vast majority (90.5%) were uncontrolled. Approximately 70% had no complications. The most common complication was diabetic nephropathy (Table 1). Attrition was the most prevalent sign of tooth wear (99.4%), with almost all subjects showing some sign, followed in order by erosion (64.8%), abrasion (31.3%), and abfraction (7.3%). Erosion and abfraction showed significant association with age group (*p* < 0.05) (Table 2). The majority of the tooth wear was of moderate to high severity. Age group was significantly associated with severity level (*p*=0.017). Mild tooth wear severity was the highest proportion found in age groups 35–44 years and 45–54 years (53.8% and 41.2%, respectively). Moderate tooth wear was the highest proportion found in age groups 55–65 years and 65–74 years (52.2% and 44.0%, respectively) as shown in Table 3. There were no significant differences in the frequencies of any of the signs of tooth wear with respect to characteristic of diabetes (Table 4).

## 6. Discussion

Our results confirm that tooth wear is highly prevalent in Thai adults and older adults. Moreover, tooth wear is very common among those with diabetes in Thailand. Studies on tooth wear in diabetic patients are rare in Thailand. As with other studies of adults, this study showed a high prevalence of tooth wear [10–13]. Similar findings were reported in a study of tooth wear in the aging population of northwest China, where the prevalence of tooth wear ranged from 85.5% in molar to 100% in canine teeth [12]. The prevalence of attrition in our study is higher than the study in the Indian subjects [11]. However, all previous studies included subjects with different age ranges than ours. This might affect the prevalence of tooth wear. When investigating the tooth wear severity, the moderate tooth wear (level 3) in our study in those 60 years and above was lower than the Dutch population who were 65–74 years (24.0% vs 82.0%) [13]. Several studies also found that the severity of tooth wear increased when age increased [13, 20, 21].

The data from our study as well as previous studies gave an implication that the prevalence of tooth wear is high and becoming an important dental public health problem. It could be due to the accumulation of certain dietary patterns, high consumption of citrus fruits, inappropriate brushing technique, and parafunctional habits [22, 23].

## 7. Limitations of the Study

Our study has some limitations. There are some limitations of the TWI index. It is a multifactorial tooth wear index; therefore, only one single code for each surface irrespective of cause. Therefore, in some tooth surfaces, there might be tooth wear due to erosion and attrition. The data could be missing. Since we did not assess the frequencies and severity of tooth wear in a sample without diabetes, we cannot say whether those with diabetes have greater tooth wear than those without diabetes. Therefore, the findings should be interpreted with caution. However, our results do suggest that tooth wear is present in those with diabetes regardless of diabetes characteristics. This suggests that it is important for the dental practitioner to evaluate periodontal disease as well as tooth wear and assist patients in developing a satisfactory diabetic diet. A diet high in raw fruits and vegetables, nuts, and unrefined grains may be difficult for those with severe tooth wear.

## 8. Conclusion

There was a high prevalence of tooth wear among diabetic patients. The role of prevention is vital in maintaining the integrity of the teeth and to avoid treating those worn teeth in diabetic patients.

## Figures and Tables

**Table 1:** Characteristics of the study population (*n*=179).

Characteristics	*n* (%)
*Sex*	
Male	61 (34.1)
Female	118 (65.9)

*Age (years)*	
35–44	13 (7.3)
45–54	51 (28.5)
55–64	90 (50.3)
65–74	25 (14.0)
Mean ± SD	56.5 ± 7.8

*Duration of having diabetes*	
Less than 1 yr	30 (16.8)
1–5 yrs	70 (39.1)
More than 5 yrs	79 (44.1)
Mean ± SD	6.5 ± 6.3

*Fast blood sugar (FBS)*	
Controlled	17 (9.5)
Uncontrolled	162 (90.5)

*Complication from diabetes*	
No complication	127 (70.9)
At least one complication	52 (29.1)

*Type of complication (n*=52)	
Diabetic retinopathy	7 (13.5)
Diabetic nephropathy	42 (80.8)
Peripheral vascular disease	6 (11.5)
Coronary vascular disease (remarks: four patients had two complications)	1 (2.0)

**Table 2:** Prevalence of tooth wear according to age group.

Age group (years)	*n*	Attrition	Erosion	Abrasion	Abfraction
		*n* (%)	*n* (%)	*n* (%)	*n* (%)
35–44	13	12 (92.3)	5 (38.5)	2 (15.4)	3 (23.1)
45–54	51	51 (100.0)	29 (56.9)	21 (41.2)	1 (2.0)
55–64	90	90 (100.0)	66 (73.3)	25 (27.8)	9 (10.0)
65–74	25	25 (100.0)	16 (64.0)	8 (32.0)	0 (0.0)
*p* value^a^		0.073	0.041	0.242	0.021

^a^Fisher exact test.

**Table 3:** Severity of different types of tooth wear according to age group (*n*=179).

Severity level	*n*	Age group				*p* value^a^
		35–44 yrs. *n* (%)	45–54 yrs. *n* (%)	55–64 yrs. *n* (%)	65–74 yrs. *n* (%)	
No tooth wear (level 0)	1	1 (7.7)	0 (0.0)	0 (0.0)	0 (0.0)	0.073
Very mild (level 1)	9	2 (15.4)	1 (2.0)	5 (5.6)	1 (4.0)	0.242
Mild (level 2)	58	7 (53.8)	21 (41.2)	23 (25.6)	7 (28.0)	0.083
Moderate (level 3)	76	2 (15.4)	16 (31.4)	47 (52.2)	11 (44.0)	0.018
Severe (level 4)	35	1(7.7)	13(25.5)	15(16.7)	6(24.0)	0.407
Total	179	13 (100.0)	51 (100.0)	90 (100.0)	25 (100.0)	0.017

^a^Fisher's exact test.

**Table 4:** Tooth wear and diabetic status.

Diabetic status		Attrition	Erosion	Abrasion	Abfraction
		*n* (%)	*n* (%)	*n* (%)	*n* (%)
*Duration of having diabetes*					
Less than 1 yr	30	29 (96.7)	19 (63.3)	12 (40.0)	2 (6.7)
1–5 yrs	70	70 (100.0)	47 (67.1)	19 (27.1)	4 (5.7)
More than 5 yrs	79	79 (100.0)	50 (63.3)	25 (31.6)	7 (8.9)
*p* value^a^		0.168	0.893	0.441	0.862

*Fast blood sugar (FBS)*					
Controlled	17	17 (100.0)	12 (70.6)	8 (47.5)	3 (17.6)
Uncontrolled	162	161 (99.4)	104 (64.2)	48 (29.6)	10 (6.2)
*p* value^a^		1.00	0.791	0.171	0.112

*Complication from diabetes*					
No complication	127	126 (99.2)	81 (63.8)	41 (32.3)	10 (7.9)
At least one complication	52	52 (100.0)	35 (67.3)	15 (28.8)	3 (5.8)
*p* value^a^		1.00	0.732	0.724	0.759

^a^Fisher's exact test.

## Data Availability

The data used to support the findings of this study are available from the corresponding author upon request.
